# Human Milk Oligosaccharide-Stimulated *Bifidobacterium* Species Contribute to Prevent Later Respiratory Tract Infections

**DOI:** 10.3390/microorganisms9091939

**Published:** 2021-09-12

**Authors:** Shaillay Kumar Dogra, Francois-Pierre Martin, Dominique Donnicola, Monique Julita, Bernard Berger, Norbert Sprenger

**Affiliations:** Nestlé Institute of Health Sciences, Société des Produits Nestlé S.A., 1000 Lausanne 26, Switzerland; ShaillayKumar.Dogra@rd.nestle.com (S.K.D.); Francois-Pierre.Martin@rd.nestle.com (F.-P.M.); dominique.donnicola@rdls.nestle.com (D.D.); monique.julita@bluewin.ch (M.J.); bernard.berger@rdls.nestle.com (B.B.)

**Keywords:** *Bifidobacterium*, acetate, fucosylated glycans, respiratory infections, bronchitis, machine learning based classification models

## Abstract

(1) Background: Human milk oligosaccharides (HMOs) may support immune protection, partly via their action on the early-life gut microbiota. Exploratory findings of a randomized placebo-controlled trial associated 2′fucosyllactose (2′FL) and lacto-N-neotetraose (LNnT) formula feeding with reduced risk for reported bronchitis and lower respiratory tract illnesses (LRTI), as well as changes in gut microbiota composition. We sought to identify putative gut microbial mechanisms linked with these clinical observations. (2) Methods: We used stool microbiota composition, metabolites including organic acids and gut health markers in several machine-learning-based classification tools related prospectively to experiencing reported bronchitis or LRTI, as compared to no reported respiratory illness. We performed preclinical epithelial barrier function modelling to add mechanistic insight to these clinical observations. (3) Results: Among the main features discriminant for infants who did not experience any reported bronchitis (*n* = 80/106) or LRTI (*n* = 70/103) were the 2-HMO formula containing 2′FL and LNnT, higher acetate, fucosylated glycans and *Bifidobacterium*, as well as lower succinate, butyrate, propionate and 5-aminovalerate, along with Carnobacteriaceae members and *Escherichia*. Acetate correlated with several *Bifidobacterium* species. By univariate analysis, infants experiencing no bronchitis or LRTI, compared with those who did, showed higher acetate (*p* < 0.007) and *B. longum* subsp. *infantis* (*p* ≤ 0.03). In vitro experiments demonstrate that 2′FL, LNnT and lacto-N-tetraose (LNT) stimulated *B. longum* subsp. *infantis* (ATCC15697) metabolic activity. Metabolites in spent culture media, primarily due to acetate, supported epithelial barrier protection. (4) Conclusions: An early-life gut ecology characterized by *Bifidobacterium*-species-driven metabolic changes partly explains the observed clinical outcomes of reduced risk for bronchitis and LRTI in infants fed a formula with HMOs. (Trial registry number NCT01715246.).

## 1. Introduction

“Birth seeds, breast milk feeds” essentially captures the very first phase of early-life microbiota development and summarizes differences in microbiota development observed by mode of delivery and diet. The infant gut is progressively and sequentially colonized with distinct microbial populations during infancy and early childhood, from an aerobic to an anaerobic milk-oriented microbiome first, then to a more diverse adult-like microbiome, reaching a stable composition at around 2 to 3 years [1,2,3]. Typically, during the first few months of life, a high *Bifidobacterium* dominance is observed before the microbiome diversifies with the introduction of a complementary weaning diet [2,4,5]. Early and frequent antibiotic use, cesarean (C-) section birth and formula feeding alter the early-life microbiome development pattern during the recommended exclusive breastfeeding period of the first 4–6 months [6,7]. This period of microbiome establishment is increasingly recognized as a critical window related to future health [8,9,10,11]. Further insights to support this have been gained from observations related to microbiome disruptors including antibiotic use, C-section birth and no or short breastfeeding that have been related to inappropriate development of immune competence, evidenced by higher risk of infections and allergies [12,13,14,15].

Human milk oligosaccharides (HMOs) are one of the major components of breast milk by mass, in order after lactose and fat. Major HMOs such as those composed of N-acetyllactosamine units attached to lactose, those containing fucose and many with sialic acid are basically missing in farmed animal milks [16]. Consequently, infant formula products manufactured from animal milks do not contain those major HMO classes, yet contain small amounts of naturally present galactosyllactoses and sialyllactoses [17,18]. Historically, HMOs were first described following the observation that breastfed and formula-fed infants are colonized with different microbes in early life [19]. In breastfed infants, specific HMOs such as 2′fucosyllactose (2′FL), lacto-N-tetraose (LNT) and lacto-N-*neo*tetraose (LNnT) are supposed to be fermented by the gut microbiota, leading to increased *Bifidobacterium* abundance and metabolic activity [20,21,22,23,24]. Breastfed infants having *Bifidobacterium breve* strains able to use FL showed the highest *Bifidobacterium* abundance and a gut environment with the highest acetate concentration. Equally, LNT and LNnT were shown to specifically boost specific *Bifidobacterium* species such as *B. longum* subsp. *infantis* or *B. breve* in preclinical models [25,26,27,28].

We recently reported exploratory findings describing the effects of 2′FL and LNnT on the early-life gut microbiota of formula-fed infants, and demonstrated that formula supplementation with these two HMOs shifted the microbiota closer to that observed in breastfed infants and was associated with reduced risk of antibiotic use later in life [29,30]. Notably, 2-HMO formula-fed infants had a significantly reduced risk of experiencing reported bronchitis and lower respiratory tract illnesses (LRTI), yet gut microbial mechanisms and markers in relation to these clinical results remain unknown [29].

To this end, we employed machine-learning-based methods [31] to identify microbiota taxa, metabolites and stool gut health indicator proteins measured at 3 months of age that associated with susceptibility to bronchitis or LRTI from 3 to 12 months of infant age. Using this approach, we generated a hypothesis on the possible mechanisms of HMO action, which were further tested.

## 2. Materials and Methods

### 2.1. Clinical Trial

The trial was registered in www.clinicaltrial.gov (accessed on 16 August 2021) with number NCT01715246 and was previously described for the full trial population [29] and for the per protocol formula-fed trial population [30] that we further explored here.

The trial population for the formula-fed groups consisted of healthy, full-term male and female infants from birth to 14 days old at enrollment, who were exclusively formula fed at the time of enrollment. Eligible infants were randomly assigned to one of two study formulas (Control or Test) using mode of delivery (vaginal or cesarean section) and gender as stratification factors (randomization was carried out using a permuted block algorithm with Medidata Balance (New York, NY, USA)). Randomized infants received exclusive feedings with the Test or Control formulas from enrollment through 4 months of age in amounts suitable for their weight, age and appetite. The Test formula was an intact protein cow’s milk protein-based infant formula with 2 HMOs (2′fucosyllactose (2′FL) and lacto-N-neotetraose (LNnT)) at 1.5 g/L and a ratio of 2:1 between the 2 HMOs. The Control formula was the same intact protein cow’s milk protein-based infant formula without the 2 HMOs, but with 1.5 g/L additional lactose.

Randomized infants were allowed the introduction of complementary weaning food and continued feedings with the Control or Test formulas through 6 months of age, and then both groups received the same intact protein cow’s-milk-based follow-up formula for feedings from 6 to 12 months of age.

The primary objective of the randomized, controlled clinical trial was to evaluate the non-inferiority of weight gain from enrollment to 4 months of age in infants fed the Test formula, compared with those fed the Control formula. Secondary objectives included the evaluation of differences between the formula groups in body weight, body length, head circumference, digestive tolerance, formula compliance, stool protein markers for intestinal status, stool microbiota, medication use and morbidity through 12 months of age. Infection-related morbidity was assessed based on an a priori-formulated hypothesis that the 2 HMOs help to prevent infections [30].

Adverse events were reported as described in Puccio et al. [29], then coded and categorized by a single physician, who was not involved in the study conduct using the Medical Dictionary for Regulatory Activities (MedDRA) System, Organ and Class (SOC) categories as well as the Preferred Terms (PTs) within each SOC category. Here, we focused on the per protocol population as described in Berger et al. [30] and on adverse events observed to be reduced with 2-HMO formula feeding [29], namely the reported PT bronchitis (up to 12 months) and the LRTI adverse event cluster (including the PTs: bronchiolitis, bronchitis, pneumonia, LRTI, LRTI viral, RSV (respiratory syncytial virus) bronchiolitis, RSV bronchitis, viral respiratory infection). For PT bronchitis, different values exist based on the incidence of occurrence (0 = 91; 1 = 22; 2 = 4; 3 = 4). Here, we considered any incidence through 12 months of age as a case (Y) and no incidence as a control (N). For PT bronchitis, we had 91 controls and 30 cases. Since we aimed to investigate the 3-month stool markers in a prognostic manner, we removed those cases who only had an incidence of PT bronchitis up to three months, but not from 3 to 12 months. This resulted in a total of 26 cases. Further, three samples from controls were removed in the modeling due to missing values and eight subjects from controls were removed as they did not finish the study, leaving us with 80 controls.

Similarly, for LRTI (up to 12 months) we had some infants with several incidences (0 = 81; 1 = 31; 2 = 5; 3 = 4). Again, we counted any number of incidences as a case (Y) and no incidence as a control (N). Thus, we had for LRTI (up to 12 months) 81 controls and 40 cases. Next, we removed cases with an incidence of LRTI only up to three months, but not from 3 to 12 months, leaving us with 33 cases. Further, three samples from controls were removed in the modeling because of missing values and 4 subjects from controls were removed as they did not finish the study, leaving us with 74 controls.

The adverse event cluster LRTI also comprises PT bronchitis. Hence, we had all 26 bronchitis cases also represented in the LRTI cases, where we had an additional seven cases. These 7 LRTI cases were part of the control PT bronchitis strata. Of the 111 infants in total, we had 78 common controls and 26 common cases for PT bronchitis and LRTI from 3 to 12 months of age.

### 2.2. Stool Microbiota Composition, Metabolites and Gut Health Markers

Stool samples were processed for microbiota composition analysis by V3 and V4 region of the 16S rRNA gene amplicon sequencing, as previously described [30]. Notably, the employed bioinformatics analysis pipeline allowed for species and subspecies identification of the genus *Bifidobacterium* [32].

The biochemical composition of the stool samples was analyzed using a well-established metabonomic approach via proton Nuclear Magnetic Resonance (^1^H NMR) Spectroscopy [33,34]. Quantitative profiles of major metabolites were acquired, including amino acids (phenylalanine, tyrosine, isoleucine), short-chain fatty acids (SCFA) (propionate, butyrate, acetate, valerate, iso-valerate, 5-amino-valerate), organic acids (lactate, succinate) and carbohydrates (fucosyl-glycans).

The gut health markers calprotectin, α1-antitrypsin and elastase were quantified by Biotecon Diagnostics GmbH (Potsdam, Germany). Quantification of α1-antitrypsin was carried out using a stool extraction kit and enzyme-linked immunosorbent assay (ELISA) provided by Immundiagnostic AG (Bensheim, Germany). Elastase was quantified using the Schebo^®^ Quick-PrepTM E1-stool extraction system (Schebo Biotech, Gießen, Germany) and the ScheBo^®^ Pancreatic Elastase 1 assay (ScheBo^®^ Biotech). Calprotectin was quantified upon extraction with the Bühlmann Smart-Prep (Dresden, Germany) stool extraction system with the EK-CAL ELISA kit (Bühlmann, Dresden, Germany).

### 2.3. In Vitro Fermentation Assay and Analysis

We used as carbohydrate sources the HMOs LNT (Lacto-N-tetraose), LNnT, 2′FL, 6′SL (6′sialyllactose) (all from Glycom A/S, Lyngby, Denmark) together with glucose and lactose (Fluka, Buchs, Switzerland), to ferment using the *B. longum* subsp. *infantis* type strain (ATCC 15697). The *B. longum* subsp. *infantis* was grown overnight in glucose-free MRS (DeMan, Rogosa and Sharpe) broth supplemented with 1% of each carbohydrate source in anaerobic conditions at 37 °C. Anaerobic conditions were achieved using BD GasPak system (Becton, Dickinson and Company, Franklin Lakes, NJ, USA). The optical density (OD) at 600 nm was measured before pelleting the bacteria by centrifugation and resuspension in PBS. To reach a final OD of 0.1, an aliquot of each preparation was added to 10 mL of DMEM cell culture media containing 0.1% glucose (Gibco™: DMEM, low glucose, GlutaMAX™ Supplement, pyruvate) further supplemented with 1% of each carbohydrate source for one night at 37 °C in anaerobic conditions. The OD and pH were measured and after centrifugation the conditioned media supernatants were sterile filtered using a 0.22 µm filter. These spent culture media were then used for further experiments and analysis.

Organic acids and short-chain fatty acids were quantified in the conditioned DMEM media using an Ultimate 3000 HPLC system (Thermo Fisher Scientific, Dionex, Sunnyvale, CA, USA) equipped with a HiPlex H analytical column (8 µm; 300 × 7.7 mm; Polymer Laboratories (Varian, London, UK)) and an U3000 RS diode array detector (Thermo Fisher Scientific) set to an absorption wavelength of 210 nm. The system was operated isocratically with 5 mM sulfuric acid at a flow rate of 0.6 mL/min and a temperature of 55 °C. Quantification was carried out using external standard curves from 0.5 to 100 mM of the respective authentic organic acids (Fluka (Sigma), Switzerland). Dionex Chromeleon software version 6.8 (Thermo Fisher Scientific) was employed to pilot the HPLC and to quantify the organic acids.

For NFkB stimulation analysis, HT−29 clone 34 cells were seeded in a 96-well plate at 2 × 10^4^ cells/well and used 2 days after 80% confluence was achieved. Culture media were replaced with fresh antibiotic-free media for 1 day and then replaced with the media containing the tested carbohydrates and conditioned media. Cells were then either challenged with 2 ng/well TNFα (Bio-Techne AG, Zug, Switzerland) or *Salmonella* SL1344 (NCTC 13347) at 10^6^ cfu/well. After 24 h at 37 °C at 5% CO_2_, the NFkB reporter luminescence was measured using the Phospha-light system (Applied Biosystems, Foster City, CA, USA) according to manufacturer’s instructions and expressed as the relative amount compared to the unconditioned glucose control.

Caco-2 (ATCC HTB-37) cells were seeded in 24-well plates at 1 × 10^5^ cells/well and cultured for 14 days at 5% CO_2_ and 37 °C before performing the adhesion/invasion assay with *Salmonella* SL 1344 (NCTC 13347). *Salmonella* SL1344 was grown to logarithmic phase in LB (Luria Bertani) broth and diluted to an OD 0.1 (approximately 1 × 10^8^ cfu/mL) in DMEM at pH 5.8. The day before the experiment, the Caco-2 cell culture media were replaced with DMEM without antibiotics. DMEM media containing the different carbohydrates and the conditioned media were adjusted to pH 5.8 and added to Caco-2 cells together with 1 × 10^7^ cfu/well *Salmonella* SL1344. Cells were incubated for 60 min. Thereafter, cells were washed 3 times with fresh culture media and incubated for another 60 min with culture media containing 100 µg/mL gentamycin. Cells were again washed 3 times with PBS (phosphate-buffered saline, Sigma, St. Louis, MO, USA) to remove gentamycin, before Caco-2 cells were lysed with sterile MilliQ-water with 1% Triton X-100 (Sigma) and plated on LB agar plates to enumerate the *Salmonella* that invaded the cells.

### 2.4. Machine Learning Algorithms and Statistical Analysis

We used sparse partial least square discriminant analysis (sPLS-DA) [35] to investigate the separation of cases and controls based on three-months-of-age stool microbiota composition, metabolites and gut health markers, as well as mode of delivery and formula type (with or without HMOs). The modeling was carried out in R [36] using the package caret [37] and mixOmics [38] using standard cross-validation methods (10-fold with 10 repeats) to avoid over-fitting of the models. The sPLS-DA modeling was run on balanced error rate (BER), which calculates the average fraction of mis-classified samples in each class, weighted by the number of samples in each class. Additionally, we used random down-sampling to balance out the two classes—cases and controls—as we had fewer cases than controls.

For statistical analysis, non-parametric tests were performed (Mann–Whitney and Kruskal–Wallis tests) using GraphPad Prism software version 8.3.0.

## 3. Results

### 3.1. Cohort Description

**Figure 1:**
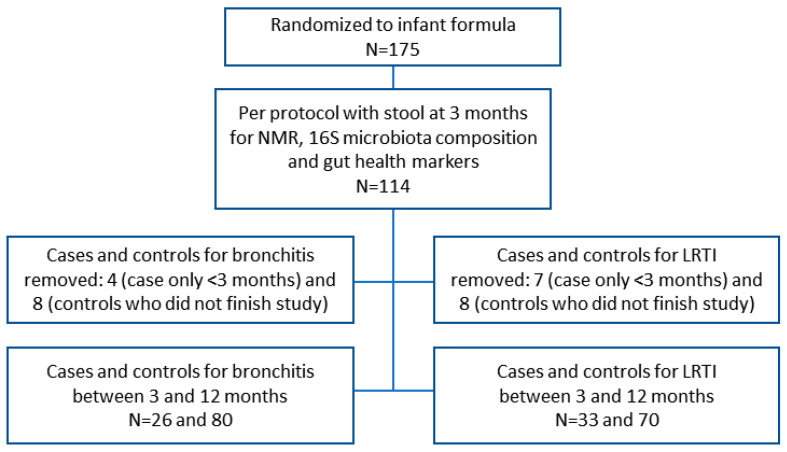
Subject flow chart showing the number of per protocol formula-fed infants with stool sample at three months of age, by controls and cases who experienced at least one reported bronchitis or lower respiratory tract infection (LRTI) between three and 12 months of age.

We sought to further explore microbiota taxa, metabolites and stool gut health indicator proteins from per protocol infants with stool samples at three months of age, and susceptibility to reported bronchitis or LRTI from 3 through 12 months of age [29,30]. Demographics and infant characteristics are shown in Table 1. The flow chart (Figure 1) depicts the number of infants randomized to the trial and the subgroup of infants available for the exploratory study reported here. The grouping of the formula-fed infants by controls and cases is equilibrated for all parameters except for their exposure to the 2-HMO-containing formula.

### 3.2. Several Microbiome and Host Features Relate to Lower Respiratory Tract Infections

We used ^1^H-NMR-identified metabolites, 16S gene-based microbiota composition (at genus and for bifidobacteria at species and subspecies level) and host gut health indicator proteins from infant stool at three months of age along with Test formula and mode of delivery to generate a total of 60 features used to categorize infants who experienced (Yes class) or did not experience (No class) bronchitis or LRTI from 3 to 12 months of age.

The class imbalance of data with controls being about three times greater as compared to cases (bronchitis: 80 controls and 26 cases; LRTI: 70 controls and 33 cases) posed a challenge, particularly for the Random Forest and Support Vector Machine algorithms. These methods required up-sampling to obtain acceptable class separation. Yet, using Random Forest with Boruta feature selection identified relative acetate, butyrate and 5-aminovalerate, together with *Lactobacillus* and succinate, as the most discriminant features (data not shown). Support vector machine combined with recursive feature elimination identified the same features together with fucosyl-glycans, lactate and *Bifidobacterium longum* subsp. *infantis* and other *Bifidobacterium* species, to name the most prominent (data not shown).

**Figure 2:**
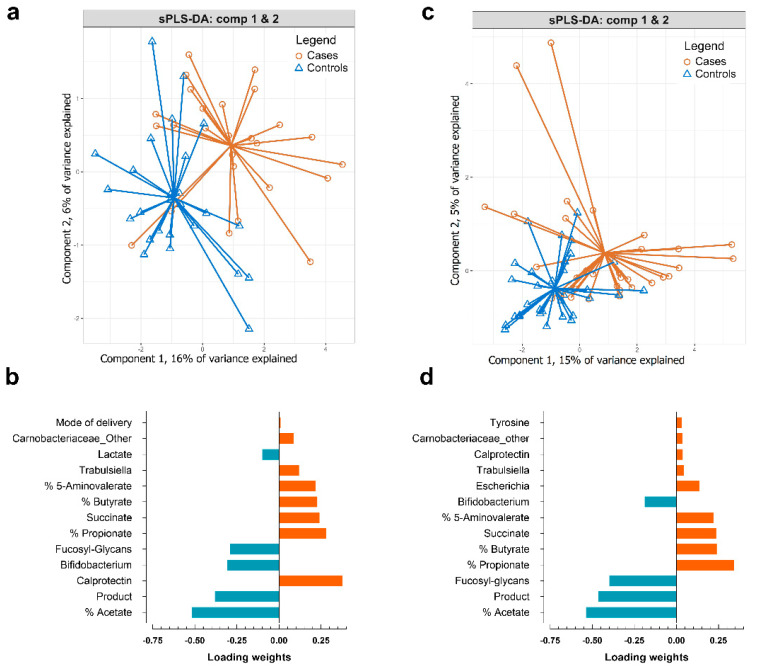
Representative sPLS-DA plots and loadings of the five independent rounds of sPLS-DA modeling with down-sampling to separate controls and cases for bronchitis (*n* = 52) (**a**,**b**) and LRTI (*n* = 66) (**c**,**d**). Results from all five sPLS-DA modeling rounds for both bronchitis and LRTI are provided in Table S1.

Classification using sPLS-DA with random down-sampling of controls was able to reasonably separate the controls from cases for bronchitis and LRTI, with a median cross validation overall accuracy, from 10-fold with 10 repeats, of about 60 to 70% and 60 to 65% across the 10 repeats for bronchitis and LRTI, respectively, with component 1 alone (Figure S1). We next focused on a more detailed analysis using five independent rounds of random down-sampling of controls for both bronchitis and LRTI. Across the rounds, the cases are always the same and it is controls that are randomly chosen, given that cases are far fewer than controls. The sensitivity–specificity receiver operating characteristic (ROC) curve with component 1 showed an area under the curve (AUC) between 0.74 (*p*-value 0.004) and 0.82 (*p* = 6.6 × 10^−5^) for bronchitis and between 0.71 (*p* = 0.003) and 0.8 (*p* = 3.7 × 10^−5^) for LRTI, across the five runs, respectively (Table S1). Class separation from representative examples of the five independent random down-sampling rounds are shown in Figure 2, together with the features of component 1 used by sPLS-DA to separate the classes for bronchitis and LRTI.

Consistently, separation of controls from bronchitis cases is driven by 2-HMO supplementation followed by fucosylated glycans, relative amount of acetate, lactate and the genus *Bifidobacterium*, with higher relative amounts in controls. Features picked by the model indicating cases in component 1 are the organic acid succinate, the relative amount of the SCFAs butyrate and 5-amino-valerate and propionate, as well as calprotectin, C-section birth, *Trabulsiella*, *Escherichia* and the family of unidentified members of the Carnobacteriaceae.

**Figure 3:**
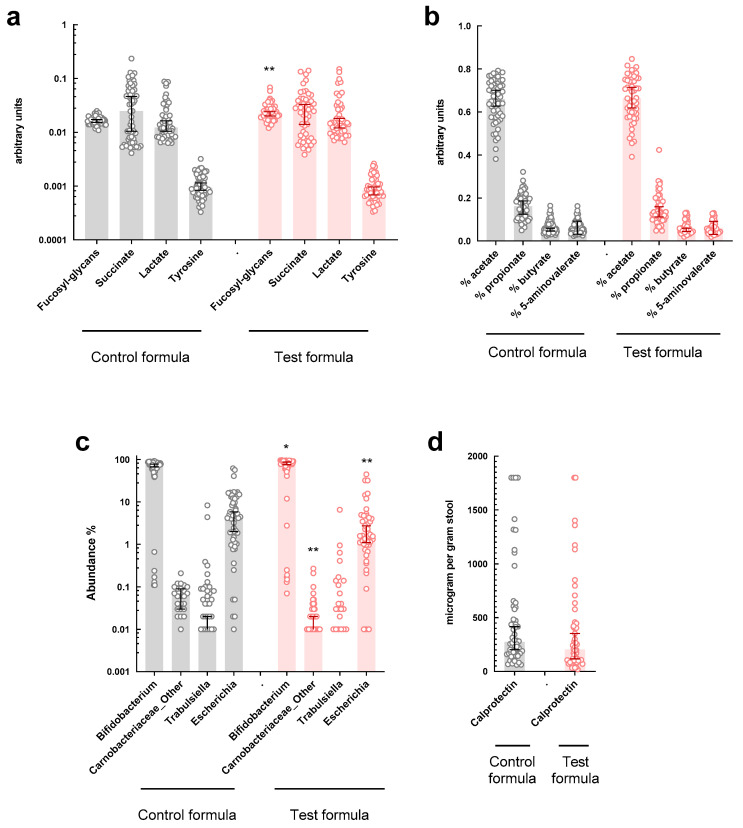
Comparison of the identified main features from the sPLS-DA model by feeding group: (**a**,**b**) relative abundance of metabolites; (**c**) relative abundance of microbial taxa; (**d**) concentration of calprotectin. (*n* = 106; * indicate *p* < 0.05, ** *p* < 0.01 by non-parametric *t*-test).

Next, we performed univariate analysis of the sPLS-DA-identified features using the full data set to compare feeding groups. We observed statistically significantly higher relative fucosyl-glycan amounts and *Bifidobacterium* abundance in feces of 2-HMO (Test) formula-fed infants, and lower abundance of the unidentified Carnobacteriaceae and *Escherichia* (Figure 3).

**Figure 4:**
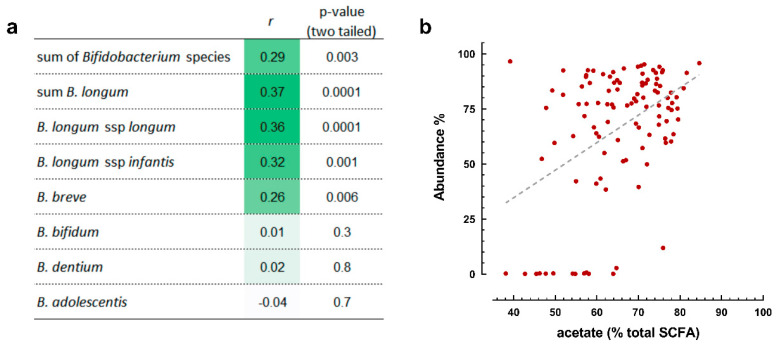
Association between relative acetate and *Bifidobacterium* species in infant stool samples. (**a**) Spearman correlation; (**b**) linear regression analysis of the sum of *Bifidobacterium* species and relative amounts of acetate.

Together, the case–control analysis indicates that an early-life gut ecology characterized by higher relative abundance of Bifidobacterium, together with higher relative acetate and lower relative butyrate, propionate and 5-aminovalerate, associates with protection from reported bronchitis and LRTI from three to 12 months of age. Therefore, we further investigated the relationship between relative acetate and the main *Bifidobacterium* species identified in our study. We observed a positive correlation between relative acetate amount in stool and the sum of all Bifidobacterium and the sum of the *B. longum* taxa together (Figure 4a). *B. longum* subsp. *longum* and *B. longum* subsp. *infantis*. *B. breve* also showed a positive although less strong correlation (Figure 4a). No statistically significant correlation was seen with *B. bifidum*, *B. dentium* and *B. adolescentis*. Similarly, the linear regression analysis illustrates a statistically significant correlation between the relative *Bifidobacterium* and acetate abundances (Figure 4b).

**Figure 5:**
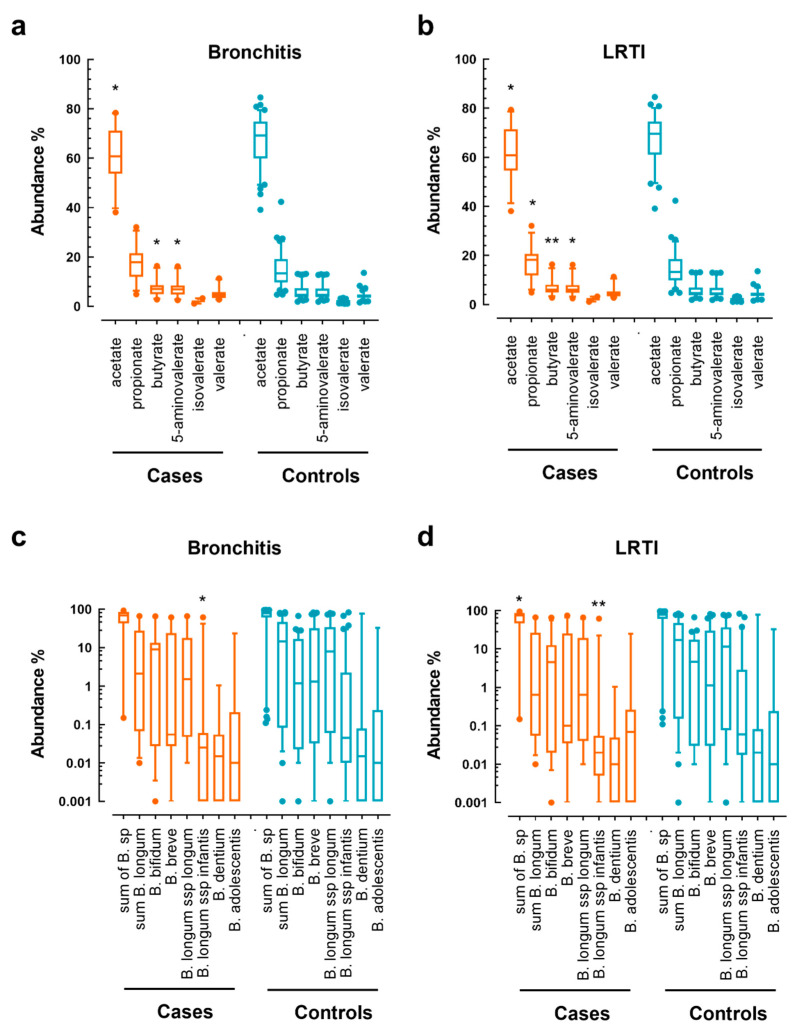
Comparison of relative amounts of short-chain fatty acids and *Bifidobacterium* species in the stool of infants at three months of age between infants who did not (controls) or did (cases) experience any bronchitis and LRTI thereafter from 3 to 12 months of age: (**a**,**b**) relative abundance of the short-chain fatty acids for bronchitis or LRTI cases and controls; (**c**,**d**) relative abundance of the *Bifidobacterium* species for bronchitis or LRTI cases and controls (* indicate *p* < 0.05, ** *p* < 0.01 by non-parametric *t*-test).

To complete the analysis with the full data set, we investigated in a univariate approach the relative content in SCFAs and *Bifidobacterium* taxa abundances, in controls and cases for both bronchitis and LRTI from 3 to 12 months of age (Figure 5). As expected from the machine-learning-based classification models, the relative acetate amount was higher in the stool samples of control infants at three months of age as compared with cases who experienced at least one bronchitis or LRTI event thereafter until 1 year of age. For butyrate and 5-aminovalerate, we observed higher relative amounts in the three-month stool samples of infants who experienced at least one bronchitis or LRTI event thereafter. Equally, for propionate, higher relative amounts were observed that reached statistical significance only for LRTI. Interestingly, only *B. longum* subsp. *infantis* was consistently higher in the stool of control infants as compared to infants who experienced bronchitis and LRTI. Yet, the *B. longum* subsp. *infantis* was only a minor contributor to the total *Bifidobacterium* population in our study infants. For LRTI, the sum of *B. longum* showed a slightly higher relative abundance in controls compared to cases.

The identified features from the clinical trial indicate an important role of bifidobacteria metabolism, especially *B. longum* subsp. *infantis* and other *Bifidobacterium* species able to ferment specific HMOs. Therefore, we further explored whether and how HMO-stimulated *Bifidobacterium* metabolites alter epithelial barrier function upon an inflammatory or pathogen challenge in an experimental model.

### 3.3. Acetate Produced by HMO Stimulated Bifidobacterium longum subsp. infantis Supports Epithelial Barrier Function

**Figure 6:**
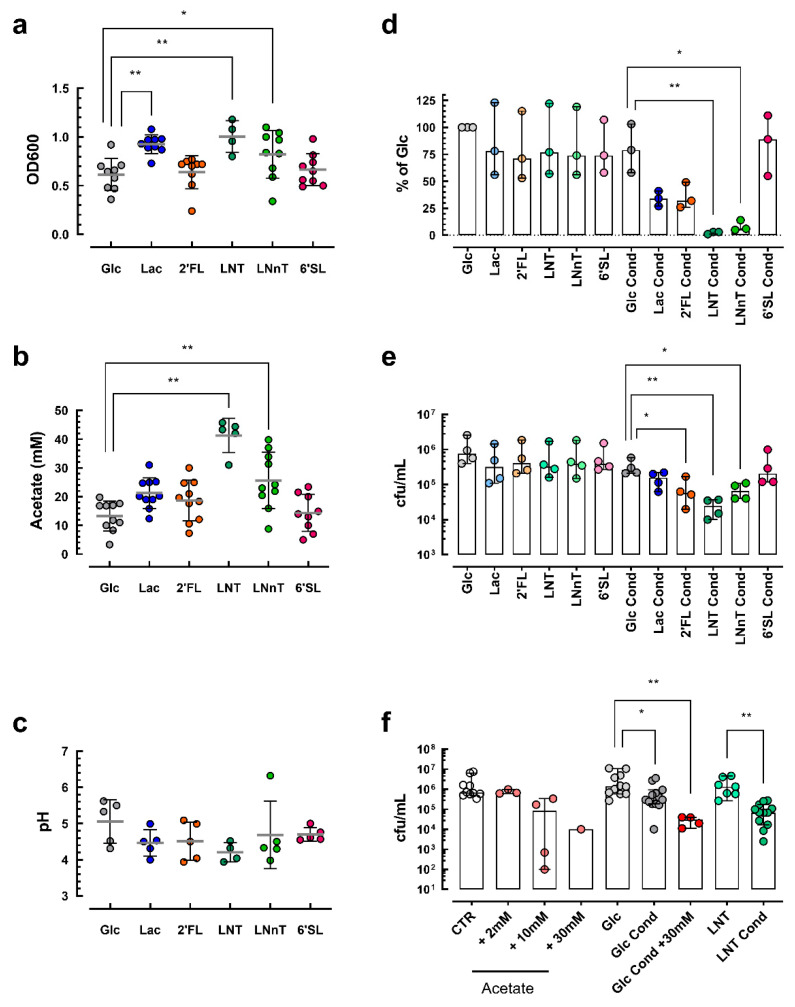
In vitro experiments with *B. longum* subsp. *infantis* conditioned with glucose (Glc), lactose (Lac), 2′fucosyllactose (2′FL), lacto-N-tetraose (LNT), lacto-N-neotetraose (LNnT) and 6′sialyllactose (6′SL): (**a**) bacterial growth monitoring using optical density at 600 nm (OD600); (**b**) quantification of acetate in spent culture media from conditioned *B. longum* subsp. *infantis*; (**c**) measurement of conditioned spent culture medium pH; (**d**) measurement of NFkB activation by TNFα in the presence of control and conditioned (cond) spent culture media; (**e**) measurement of *Salmonella* invasion in cultured epithelial cells in the presence of control and conditioned spent culture media; (**f**) measurement of *Salmonella* invasion with different amounts of acetate added to fresh or spent culture media in comparison to control and spent culture media. Symbols stand for independent experiments each with 3 replicates (* *p* < 0.05, ** *p* < 0.01 by Kruskal–Wallis or non-parametric *t*-test).

*B. longum* subsp. *infantis* (ATCC 15697) were grown in the presence of additional glucose, lactose or the HMOs 2′FL, LNnT, LNT or 6′SL. LNT was assessed due to its structural similarity to LNnT and 6′SL, as it is structurally very different from the other HMOs utilized. *B. longum* subsp. *infantis* grew on all substrates, with lactose and LNT generating the highest bacterial growth (Figure 6a). In terms of metabolic activity, we found the highest amounts of acetate in the spent culture media from *B. longum* subsp. *infantis* conditioned with LNT and LNnT as substrates (Figure 6b). No changes in pH were observed (Figure 6c). Moreover, spent culture media from *B. longum* subsp. *infantis* conditioned with LNT or LNnT strongly reduced TNFα- and Salmonella SL1344 (data not shown)-mediated NFkB activation (Figure 6d). Invasion of *Salmonella* in Caco-2 cells was significantly reduced by spent culture media from *B. longum* subsp. *infantis* conditioned with 2′FL, LNT and LNnT (Figure 6e). Since acetate was particularly increased in spent culture media from *B. longum* subsp. *infantis* conditioned with LNT and LNnT, we tested whether acetate alone was sufficient to mediate the observed epithelial barrier protection. Acetate dose dependently reduced *Salmonella* SL1344 invasion and importantly, the addition of acetate to spent culture media from *B. longum* subsp. *infantis* conditioned with glucose led to equivalent protection from *Salmonella* SL1344 as compared to spent culture media from *B. longum* subsp. *infantis* conditioned with LNT (Figure 6f). Hence, acetate is a key Bifidobacterium-produced metabolite stimulated by specific HMOs, such as LNT and LNnT, that promote epithelial barrier protection.

## 4. Discussion

The developing gut microbiome during early life plays a key role in long-term health through its action on mucosal barrier function and immune development [10,22,39]. Yet, the age-specific role of key microbial taxa dominating the early-life gut ecosystem as well as key environmental and nutritional factors influencing their activity remains unclear. Here, we used a clinical intervention trial with 2-HMO and control formula-fed infants to identify early-life infant parameters and microbiome features related to subsequent reported LRTI, including bronchitis. We identified that feeding with a 2-HMO formula, associated with reduced risk for reported bronchitis and LRTI, is characterized by a distinct gut microbial and metabolite phenotype. This phenotype is composed of higher stool acetate, fucosyl-glycans and *Bifidobacterium* species abundance, along with lower content in butyrate, propionate, 5-aminovalerate and succinate, and lower *Escherichia* species abundance. Among these features, relative acetate and *B. longum* subsp. *infantis* abundance were higher in the stool of infants with no reported bronchitis or LRTI, suggesting an association with protection against respiratory tract infection. Our in vitro experiments further corroborate these findings, through the demonstration of metabolic activity from HMO-conditioned *Bifidobacterium longum* subsp. *infantis*, and especially the acetate production that enhanced epithelial barrier function in cell culture models.

The short-chain fatty acid acetate is a key metabolite of bifidobacteria [40], and in our study we observed a significant correlation between these two features. Additionally, by univariate analysis we observed significant differences in relative acetate and *B. longum* subsp. *infantis* measured in stool at three months of age when comparing infants who experienced bronchitis or LRTI thereafter or did not. From the correlation matrix between bifidobacteria and acetate, we can assume that other *Bifidobacterium* species such as *B. longum* subsp. *longum* and *B. breve* also contributed to the higher in vivo production of acetate. All those species are known to have strains that can use specific HMOs as substrate [19,40,41,42]. Notably, HMO utilization capacity by bifidobacteria was previously shown to lead to higher *Bifidobacterium* species abundance, with higher acetate concentration and lower stool pH [19].

In our study, *B. longum* subsp. *infantis* was associated with protection against respiratory infection in early life. Therefore, we decided to use a *B. longum* subsp. *infantis* well adapted to use HMOs [43,44] to further explore causality using a series of in vitro cell culture models. First, we confirmed previous observations, indicating that specific HMOs, mainly LNT, LNnT and 2′FL, but not 6′SL, stimulated *B. longum* subsp. *infantis* growth and metabolic activity. Second, we showed that the generated metabolic phenotype of stimulated *B. longum* subsp. *infantis* reduced NFkB activation and protected epithelial cells from invasion by *Salmonella*. Third, we demonstrate that acetate is sufficient to provide the epithelial cell protection. Hence, HMO-stimulated *B. longum* subsp. *infantis* type strain exerts epithelial barrier protection. In a series of elegant preclinical animal studies, acetate from bifidobacteria was shown to mediate protection from enterohaemorrhagic *E. coli* [45]. The authors speculate that acetate improved the epithelial barrier function, thereby providing enhanced protection from the pathogenic *E. coli*. Recently, acetate was also shown to drive gut secretory IgA production and specificity [46], which provides a mechanistic angle. The epithelial barrier protective effect seen for acetate is not confined to the intestine and provides protection via the gut–lung axis [47]. In adults, colonic-administration ^13^C-SCFA showed the highest systemic availability for acetate (36%) compared to propionate (9%) and butyrate (2%), indicating that acetate can mediate beneficial effects at other body sites [48]. In a mouse model, acetate was shown to protect from RSV, a major cause of LRTI in infants [49]. Acetate-mediated activation of lung epithelial Gpr43 reduced the cytotoxic effects of RSV and stimulated an interferon-mediated anti-viral response [49]. In another animal model, acetate was further shown to contribute to protection from bacterial superinfection of the lungs following influenza virus infection through the activation of alveolar macrophages [50]. Although bifidobacteria-produced acetate in early life may not be solely responsible, our findings suggest a likely contribution to infant protection from LRTI later in life.

The early-life gut ecology with higher relative acetate and specific bifidobacteria in infants who did not experience any bronchitis or LRTI through 12 months contrasts that of infants who did experience any bronchitis or LRTI, which is characterized by higher relative butyrate, propionate and 5-aminovalerate and succinate. This observation indicates that an age-appropriate gut microbiome development may be important for protection against respiratory infection. In a recent study, Tsukuda et al. reported SCFA and other organic acid profiles in stool samples during the first 2 years of age from a small cohort of mainly breastfed and vaginally born infants [51]. An early succinate peak was paralleled by increasing acetate, while propionate and butyrate increased with cessation of breastfeeding. This is consistent with the general observation of the development of gut microbiota diversity and composition, dominated by bifidobacteria in early life and diversifying rapidly with complementary diet [6,52]. A delay in the bifidobacteria dominance is generally observed with cesarean-section-born infants [5,53], and a recent study linked such a delay together with further alterations in the gut microbiota, leading to higher risk for respiratory infections [12]. Thus, an acetate- and bifidobacteria-dominated gut ecology during the first few months of life seems important for protection against respiratory infection, and specific HMOs support this protection.

While important biological functions leading to enhanced protection can be attributed to acetate, a bifidobacteria-dominated gut ecology associated with other biochemical molecules may also be of importance for host physiology and immune development. An important group of molecules are those derived from the aromatic amino acids tryptophan, phenylalanine and tyrosine by HMO-utilizing *Bifidobacterium* metabolism; for example, the aromatic lactic acids indolelactate, phenyllactate and 4-hydroxyphenyllactate [22,54]. Indolelactate has been shown to activate the aryl-hydrocarbon receptor. This is of particular interest, as aryl-hydrocarbon receptor signaling is important for intestinal homeostasis, barrier function in gut, skin and lungs and immunity [55,56]. Specifically, *B. longum* subsp. *infantis* supplementation to breastfed infants silenced Th2 and Th17 cytokines and induced interferon beta by an indolelactate-mediated upregulation of galectin-1 [22]. Hence, several metabolites from key HMO-utilizing bifidobacteria act through different molecular routes to strengthen protection against infection.

Using machine learning approaches, we robustly identified other features together with acetate that may contribute to enhanced protection. By our ^1^H NMR metabolomics approach, the signals integrated as fucosyl-glycans do not allow any exact structural annotation due to the complex biological mixture represented in feces. Yet, through spiking experiments we have confirmed that the ^1^H NMR signal does not correspond to 2′FL, which was part of the 2-HMO formula ingredients. However, among the identified “fucosyl-glycan” are signals of free fucose and fucose bound to other unidentified glycans. It is possible that part of the fucosyl-glycan signals stem from mucous glycans and may indicate gut mucosal fucosylation changes. Free fucose liberated from 2′FL upon microbial action may have further strengthened mucosal barrier function, possibly through upregulation of the fucosyltransferase 2 activity, as recently elaborated in preclinical models [57].

The exploratory approach we took identified several features indicating different possible early-life-gut-ecology-mediated mechanisms related to later protection from respiratory infection. Yet, study limitations must be emphasized here. Besides the limitations of the trial [1,2], primarily related to the reporting of respiratory illnesses as part of the adverse event reporting, our case–control classification approach suffered from a relatively low number of cases versus controls and from a relatively low number of subjects and features. With such limitations, some machine learning classification algorithms do not perform at their best. Therefore, we initially explored the data using multiple classification methods (Random Forests with Boruta feature selection, Support Vector Machines [58] and sPLS-DA), all based on different algorithms, and observed some features that were consistently selected by the different methods. Among them are the relative acetate amounts, fucosyl-glycans, product and relative butyrate and succinate. For the detailed analysis presented here, we chose to use sPLS-DA. To overcome the class imbalance, we ran the sPLS-DA classification in five independent runs by randomly selecting samples from controls to balance with the cases and found moderate separation, with the primary purpose of hypothesis generation.

## 5. Conclusions

Machine-learning-based classification proved useful to identify possible mechanisms relating early-life gut ecology to subsequent respiratory infection using data from a well-stratified and controlled intervention trial with formula-fed infants. Among the main identified features discriminating between cases and controls for early-life respiratory infections were the 2-HMO formula and the relative stool acetate, butyrate and 5-aminovalerate, as well as succinate and fucosyl-glycans. Together, these indicate the importance of a distinguished early-life gut ecology with higher relative acetate, mediated by HMO-utilizing bifidobacteria for protection against respiratory infection. This hypothesis merits further investigation, including additional detailed metabolite analysis and functional assays to assess their contribution to appropriate immune maturation during this critical early-life window.

## Figures and Tables

**Table 1:** Subject characteristics by case for bronchitis and LRTI and respective controls *.

Infant Characteristics	Cases with Bronchitis	Controls without Bronchitis	Cases with LRTI	Controls without LRTI	Cases vs. Controls Bronchitis *p*-Value	Cases vs. Controls LRTI *p*-Value
	(*n* = 26)	(*n* = 80)	(*n* = 33)	(*n* = 70)		
Formula with HMOs (*n*, % yes)	6 (23)	44 (55)	10 (30%)	38 (54)	*p* = 0.006	*p* = 0.033
Sex (*n*, % male)	18 (69)	38 (48)	33 (52.4)	29 (50.0)	ns	ns
Gestational age, wk ^#^	39.3 ± 1.0	39.2 ± 1.1	39.2 ± 1.0	39.2 ± 1.1	ns	ns
Siblings (*n*, % yes)	17 (65)	49 (61)	21 (64)	42 (60)	ns	ns
C-section (*n*, % yes)	12 (46)	29 (36)	15 (45)	24 (34)	ns	ns
Weight at birth, kg ^#^	3.4 ± 0.32	3.4 ± 0.44	3.4 ± 0.33	3.4 ± 0.44	ns	ns
Length at birth, cm ^#^	50.2 ± 1.46	49.9 ± 1.85	50.2 ± 1.60	49.8 ± 1.79	ns	ns

^#^ Values are mean ± standard deviation unless otherwise noted; * parameters by Test and Control feeding group were previously reported [29,30]. For categorical data, difference by two-tailed Chi-square test with Fisher exact probability test; for numerical data, by *t*-test. ns, not significant; Cases, from 3 to 12 months of age, any bronchitis or LRTI; Controls, no bronchitis or LRTI through 12 months of age; LRTI, lower respiratory tract infection adverse events cluster.

## Data Availability

The microbiota data presented in this study were previously reported [30] and are openly available in SRA under accession number SRP151522.

## Supplementary Materials

The following are available online at https://www.mdpi.com/article/10.3390/microorganisms9091939/s1, Figure S1: Representative sPLS-DA model accuracy to separate cases from controls, Table S1: Loadings of 5 independent rounds of sPLS-DA modeling to separate controls and cases for bronchitis (*n* = 52) and LRTI.

## Abbreviations

HMO, human milk oligosaccharide; 2′FL, 2′fucosyllactose; LNnT, lacto-N-neotetraose; LNT, lacto-N-tetraose; 6′SL, 6′sialyllactose; C-section, cesarean section; LRTI, lower respiratory tract infections; SCFA, short-chain fatty acids; ELISA, enzyme-linked immunosorbent assay; sPLS-DA, sparse partial least square discriminant analysis.
