# Supplementary material for: Human Milk Oligosaccharide-Stimulated Bifidobacterium Species Contribute to Prevent Later Respiratory Tract Infections
Source: Microorganisms. 2021 Sep 12;9(9):1939. doi: 10.3390/microorganisms9091939 (PMC8465161; doi:10.3390/microorganisms9091939)
Supplement: Supplementary file 1 [file microorganisms-09-01939-s001.zip › microorganisms-1362975-supplementary.pdf]

microorganisms

MDPI

Article

Human Milk Oligosaccharide-Stimulated *Bifidobacterium* Species Contribute to Prevent Later Respiratory Tract Infections

Shaillay Kumar Dogra, Francois-Pierre Martin, Dominique Donnicola, Monique Julita †, Bernard Berger and Norbert Sprenger *

Nestlé Institute of Health Sciences, Société des Produits Nestlé S.A., 1000 Lausanne 26, Switzerland; ShaillayKumar.Dogra@rd.nestle.com (S.K.D.); Francois-Pierre.Martin@rd.nestle.com (F.-P.M.); dominique.donnicola@rdls.nestle.com (D.D.); bernard.berger@rdls.nestle.com (B.B.)
* Correspondence: Norbert.sprenger@rdls.nestle.com; Tel.: +41−21−7858−532
† Retired.

Table S1. Loadings of 5 independent rounds of sPLSDA modeling to separate controls and cases for bronchitis (*n* = 52) and LRTI.

Bronchitis case-control

Component 1	round 1		round 2		round 3		**round 4**		round 5		**mean**
**Feature**	**value**	**rank**	**value**	**rank**	**value**	**rank**	**value**	**rank**	**value**	**rank**	**rank**
% Acetate	-0.453	2	-0.506	1			-0.516	1	-0.456	2	1.5
Product	-0.314	4	-0.449	3	-0.326	4	-0.381	2	-0.463	1	2.8
Lactobacillus					0.392	3					3.0
Bifidobacterium							-0.307	4			4.0
% Valerate									0.381	4	4.0
Fucosyl-Glycans	-0.266	6	-0.388	4	-0.616	1	-0.290	5	-0.155	8	4.8
Succinate	0.456	1	0.178	6	0.442	2	0.240	7	0.138	9	5.0
% Butyrate	0.320	3	0.057	10	0.250	5	0.227	8	0.388	3	5.8
% Propionate	0.134	12	0.489	2			0.281	6			6.7
Enterococcus	-0.242	7									7.0
% 5-Aminovalerate	0.307	5	0.045	11	0.232	6	0.217	9	0.370	5	7.2
Tyrosine	0.146	11	0.266	5							8.0
Isoleucine			0.114	8							8.0
Trabulsiella							0.119	10	0.268	6	8.0
AAT	-0.179	9	-0.158	7	-0.171	7			-0.070	10	8.3
Calprotectin	0.004	16			0.138	8	0.378	3			9.0
Phenylalanine			0.074	9							9.0
Escherichia	0.238	8	0.033	12	0.025	9					9.7
Elastase	-0.156	10									10.0
Enterobacteriaceae_Other					-0.020	10					10.0
Mode of delivery							0.009	13	0.160	7	10.0
Lactate			-0.003	13			-0.099	11			12.0
Carnobacteriaceae_Other							0.088	12			12.0
Neopterin	-0.075	13									13.0
Ruminococcus	0.046	14									14.0
Streptococcus	-0.041	15									15.0

**AUC-ROC comp 1**	0.78	0.76	0.82	0.80	0.74
**p-value**	0.0005423	0.001198	6.62E-05	0.0002183	0.003409

Component 2	round 1		round 2		round 3		round 4		round 5		**mean**
**Feature**	**value**	**rank**	**value**	**rank**	**value**	**rank**	**value**	**rank**	**value**	**rank**	**rank**
Elastase	-0.949	1					-0.911	1			1
Lipocalin					-0.966	1					1
Bifidobacterium									0.8797	1	1
Veillonella			-0.861	1			-0.409	2			2
Neopterin	-0.23	2									2
Enterobacteriaceae_Other					-0.241	2			-0.338	2	2
AAT	-0.216	3	-0.435	2	-0.092	3					3
total scfa			-0.263	3							3
Calprotectin							0.061	3			3
Lactate									0.3339	3	3

**AUC-ROC comp 1+2**	0.87	0.82	0.85	0.89	0.81
**p-value**	6.17E-06	7.15E-05	1.57E-05	1.63E-06	0.0001127

direction of cases   direction of controls

LRTI case-control

Component 1	**round 1**		round 2		round 3		round 4		round 5		**mean**
**Feature**	**value**	**rank**	**value**	**rank**	**value**	**rank**	**value**	**rank**	**value**	**rank**	**rank**
Product	-0.463	2	-0.404	3	-0.555	1	-0.477	1	-0.375	2	1.8
% Acetate	-0.537	1	-0.468	2	-0.540	2	-0.351	3	-0.372	3	2.2
Fucosyl-glycans	-0.399	3	-0.218	5	-0.425	3	-0.444	2	-0.281	5	3.6
% Propionate	0.342	4	0.351	4	0.307	4	0.272	5	0.430	1	3.6
AAT			-0.106	7							7.0
Succinate	0.236	6	0.638	1	0.051	9	0.247	6	0.091	14	7.2
% Butyrate	0.240	5	0.013	10	0.215	5	0.184	9			7.3
Actinomyces					0.089	8	0.187	8	0.261	6	7.3
% 5-Aminovalerate	0.220	7			0.198	6	0.176	10			7.7
Carnobacteriaceae_other	0.037	12					0.282	4			8.0
Enterobacteriaceae_Other									0.217	8	8.0
Escherichia	0.137	9	0.105	8			0.164	12	0.231	7	9.0
Isoleucine									0.213	9	9.0
Bifidobacterium	-0.189	8					-0.209	7	-0.155	13	9.3
Trabulsiella	0.045	10			0.172	7	0.172	11	0.202	10	9.5
Enterococcus			-0.128	6			-0.121	13			9.5
% Valerate			0.043	9	0.011	10					9.5
Tyrosine							0.054	16	0.317	4	10.0
Phenylalanine									0.197	11	11.0
Ruminococcus									0.175	12	12.0
Tyrosine	0.032	13									13.0
Veillonella							-0.093	14			14.0
Calprotectin	0.038	11					0.039	18			14.5
Mode of Delivery							0.087	15			15.0
Streptococcaceae_Other									0.002	15	15.0
Lactate							-0.044	17			17.0

**AUC-ROC comp 1**	0.78	0.80	0.71	0.77	0.74
**p-value**	0.0001226	3.74E-05	0.003385	0.0001672	0.0007977

Component 2	round 1		round 2		round 3		round 4		round 5		**mean**
**Feature**	**value**	**rank**	**value**	**rank**	**value**	**rank**	**value**	**rank**	**value**	**rank**	**rank**
Neopterin			-0.695	1							1
Veillonella					-0.968	1	-0.766	1	-0.722	1	1
Lactate	0.9432	1							0.6775	2	2
Streptococcus	0.3204	2									2
Lactate			0.6792	2							2
Phenylalanine			-0.236	3	-0.226	2	-0.462	2			2
AlphaAT	-0.088	3									3
% Valerate									0.1418	3	3
Calprotectin							0.113	4			4
Streptococcaceae_Other							-0.005	5			5
Bifidobacterium					0.1114	3					3
Enterococcus							-0.432	3			3

**AUC-ROC comp 1+2**	0.84	0.87	0.76	0.81	0.76
**p-value**	2.02E-06	3.21E-07	3.38E-04	1.26E-05	0.0002508

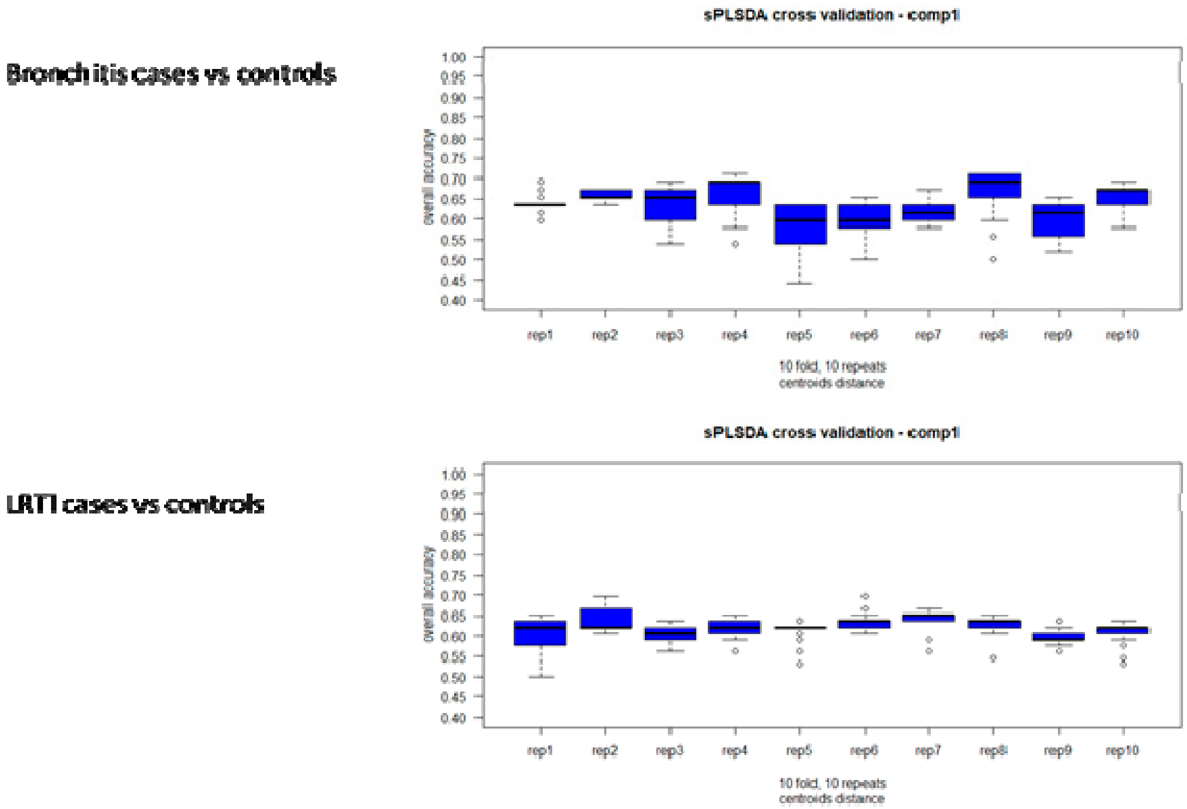

Figure S1. Representative sPLS-DA model accuracy to separate cases from controls.
